# Genes and Pseudogenes: Complexity of the RCCX Locus and Disease

**DOI:** 10.3389/fendo.2021.709758

**Published:** 2021-07-30

**Authors:** Cinzia Carrozza, Laura Foca, Elisa De Paolis, Paola Concolino

**Affiliations:** ^1^Dipartimento di Scienze di Laboratorio e Infettivologiche, UOC Chimica, Biochimica e Biologia Molecolare Clinica, Fondazione Policlinico Universitario “Agostino Gemelli” IRCCS, Roma, Italy; ^2^Dipartimento di Scienze Biotecnologiche di base, Cliniche Intensivologiche e Perioperatorie, Università Cattolica del Sacro Cuore, Roma, Italy

**Keywords:** RCCX, haplotypes, Congenital Adrenal Hyperplasia (CAH), CAH-X, Copy Number Variation (CNV), Complement Component C4

## Abstract

Copy Number Variations (CNVs) account for a large proportion of human genome and are a primary contributor to human phenotypic variation, in addition to being the molecular basis of a wide spectrum of disease. Multiallelic CNVs represent a considerable fraction of large CNVs and are strictly related to segmental duplications according to their prevalent duplicate alleles. RCCX CNV is a complex, multiallelic and tandem CNV located in the major histocompatibility complex (MHC) class III region. RCCX structure is typically defined by the copy number of a DNA segment containing a series of genes – the serine/threonine kinase 19 (*STK19*), the complement 4 (*C4*), the steroid 21-hydroxylase (*CYP21*), and the tenascin-X (*TNX*) – lie close to each other. In the Caucasian population, the most common RCCX haplotype (69%) consists of two segments containing the genes *STK19-C4A-CYP21A1P-TNXA-STK19B-C4B-CYP21A2-TNXB*, with a telomere-to-centromere orientation. Nonallelic homologous recombination (NAHR) plays a key role into the RCCX genetic diversity: unequal crossover facilitates large structural rearrangements and copy number changes, whereas gene conversion mediates relatively short sequence transfers. The results of these events increased the RCCX genetic diversity and are responsible of specific human diseases. This review provides an overview on RCCX complexity pointing out the molecular bases of Congenital Adrenal Hyperplasia (CAH) due to CYP21A2 deficiency, CAH-X Syndrome and disorders related to CNV of complement component C4.

## Introduction

Germline Copy Number Variation (CNV) is regarded as a particular DNA fragment with variable copies compared to a reference genome and primarily includes genome duplications and deletions (1). CNVs account for a large proportion of human genome (2), greatly influence cellular phenotypes such as gene expression (3), and are accountable for a plethora of diseases, in addition to representing relevant disease risk factors (4, 5). These observations raise the possibility that CNVs could be a primary contributor to human phenotypic variation and consequently evolve under selective pressures (5). Four major mechanisms have been proposed as contributors to the generation of most CNVs, including nonallelic homologous recombination (NAHR), nonhomologous end-joining, fork stalling and template switching, and L1-mediated retrotransposition (4). Multiallelic CNVs constitute a considerable fraction of large CNVs and are strictly related to segmental duplications according to their prevalent duplicate alleles (6, 7). CNVs alleles with large, homologous, and tandem repeats are susceptible to rearrangements *via* NAHR mechanism (8) such as unequal crossover (9) and gene conversion (10). In this Review, we focus on the genetic complexity of the RCCX CNV discussing the molecular bases of related human diseases as Congenital Adrenal Hyperplasia (CAH).

## *RCCX* CNV

**Figure 1:**
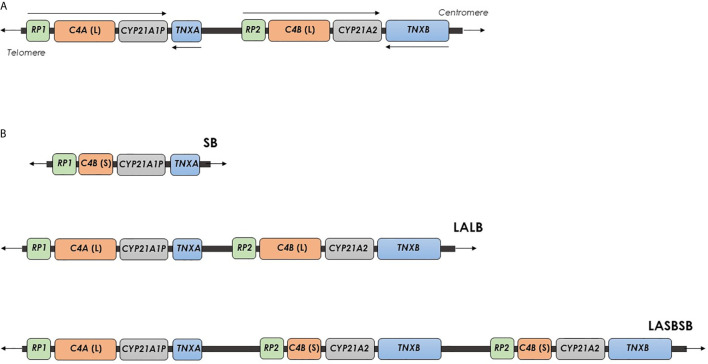
Organization of the human *RCCX* CNV on chromosome 6. **(A)** *RCCX* structure with two segments containing the genes *STK19-C4A-CYP21A1P-TNXA-STK19B-C4B-CYP21A2-TNXB*, with a telomere-to-centromere orientation. Arrows indicates the transcriptional orientation of genes. **(B)** *RCCX* structures with one, two and three segments. Each segment is indicated with two letters, the first represents the allele of the *HERV-K*(*C4*) CNV [L, Long allele (insertion) or S, Short allele (deletion)], and the second indicates the types of *C4* gene (A or B). SB, A *RCCX* structure with one segment including a *C4B* gene without *HERV-K*(*C4*) insertion. LALB, A *RCCX* structure with two segments including two *C4* genes, A and B, both with *HERV-K*(*C4*) insertion. LA SB SB: A *RCCX* structure with three segments including a *C4A* gene with *HERV*-K(*C4*) insertion and two *C4B* genes without *HERV-K(C4*) insertion.

*RCCX* CNV is a complex, multiallelic and tandem CNV located in the major histocompatibility complex (MHC) class III region (11, 12). It is an haplotypic structure typically defined by the copy number of a DNA segment containing a series of genes that lie close to each other: the serine/threonine kinase 19 (*STK19*), the complement 4 (*C4*), the steroid 21-hydroxylase (*CYP21*), and the tenascin-X (*TNX*) genes (13). *RCCX* CNV alleles commonly consist of one, two or three segments with the prevalence of approximately 17%, 69% and 14% in the Caucasian population (14). The **Figure 1A** shows the structure of the *RCCX* haplotype with two segments with the genes oriented as: *STK19*-*C4A*-*CYP21A1P*-*TNXA*-*STK19B*-*C4B*-*CYP21A2*-*TNXB* (15). *STK19* gene (originally called *G11* or *RP*), just upstream from *C4A*, encodes a nuclear Serine/Threonine Kinase protein recently identified as a regulator of NRAS activity (16–20). *STK19B*, immediately upstream from the *C4B* gene, consists only of 914 bases of the 3’ end of the original gene because the *C4*/*CYP21*/*TNX* locus duplication caused the lost of a large part of the coding DNA in this region (14, 15). *C4A* and *C4B* genes encode the two isoforms of the fourth component of serum complement (C4), an essential element for the effector arm of the humoral immune response (21). Each human *C4* gene contains 41 exons, and the gene size shows a dichotomous size variation between ~22 kb and 16 kb. The longer gene is the result of the integration of the endogenous retrovirus *HERV-K*(*C4*) into intron 9 (22). Both the *C4A* and *C4B* 3’ ends lie only 2466 bp upstream the *CYP21A1P* and *CYP21A2* transcriptional start sites, respectively. In addition, the promoter regions of *CYP21* genes are located in the *C4* intron 35 (23). *CYP21A2* gene encodes the steroid 21-hydroxylase enzyme (cytochrome P450c21), uniquely expressed in adrenal cortex, responsible for the biosynthesis of the two principal steroid hormones, aldosterone and cortisol. Both the *CYP21A2* functional gene and the *CYP21A1P* pseudogene consist in a total of ten exons spanning 3.4 kb. Sequence identity of 98% and approximately 96% characterizes their exons and intronic regions, respectively (24, 25).

With respect to the *C4* and *CYP21*, both the *TNXA* and *TNXB* genes are located in the opposite DNA strand with, consequently, an opposite transcriptional orientation. These genes partially overlap the 3’ ends of the *CYP21* genes: the last exon of *TNXA* and *TNXB* lies within the 3’ untranslated region of exon 10 in *CYP21A1P* and *CYP21A2*, respectively, and contain fibronectin type III repeats (26, 27). *TNXB* gene, encoding the extracellular matrix protein TNX, consists of 68.2 kb of DNA and includes 44 exons (28). The *TNXB* gene appears to be unique in having both its 5’ and 3’ ends buried in other genes. In fact, several start sites located into or near the *CREB-RP* gene are responsible for the *TNXB* transcription initiation. The *CREB-RP* gene lie immediately upstream of *TNXB* and encoding a protein related to the CREB transcription factor (29, 30). *TNXA* is a duplicated section of *TNXB* and consists in a truncated pseudogene containing a 120 bp deletion that causes a frameshift and a premature stop codon that render the gene non-functional (31).

An haplotypic *RCCX* CNV structure is traditionally described by the copy number of the repeated segment of *RCCX* CNV (CNV allele), and, per segment, by the alleles of HERV-K(C4) CNV and the type of *C4* gene (13). Usually, a *RCCX* segment is indicated with two letters, the first representing the alleles of the *HERV-K*(*C4*) CNV [L: long allele (insertion allele) or S: short allele (deletion allele)] and the second indicating the type of *C4* gene (A or B). The multiplication of these two letters indicates the presence of two and three segments (**Figure 1B**) (11, 13). Very rare *RCCX* CNV alleles with four segments have been also reported (32, 33). In addition, in order to define the exact structure (presence or absence of *HERV-K*(*C4*) insertion and type of *C4* gene) of a *RCCX* CNV, specific molecular approaches have been proposed (11, 34).

## *RCCX*-Associated Diseases

The genetic diversity of the *RCCX* is highly attributable to NAHR: unequal crossover facilitates large structural rearrangements and copy number changes, whereas gene conversion mediates relatively short sequence transfers (9, 10). The results of these events increase the *RCCX* genetic diversity and are responsible of specific human diseases.

### CAH Due to 21-Hydroxylase Deficiency

CAH is a group of genetic autosomal recessive disorders that affects adrenal steroidogenesis in the adrenal cortex. The vast majority of the CAH cases, approximately 95%, are related to 21-hydroxylase deficiency due to pathogenic variants accounted in *CYP21A2* gene. 21-hydroxylase enzyme is responsible for the conversion of 17-hydroxyprogesterone to 11-deoxycortisol and progesterone to deoxycorticosterone (35, 36). The impairment of cortisol and aldosterone production is directly related to the clinical form of the disease that ranges from classic (CL) or severe to non-classic (NC) or mild late onset (37, 38). As above-mentioned, both the *CYP21A2* gene and its *CYP21A1P* pseudogene are composed by a total of 10 exons, sharing a high rate of homology (25, 39). The *CYP21A1P* pseudogene is inactivated by multiple deleterious variants (small insertions/deletions and point pathogenic variants) responsible for the synthesis of a non-functional protein. Intergenic recombination events represent more than 95% of deleterious variants leading to 21-hydroxylase deficiency. Approximately 75% of the deleterious variants are transferred by small conversions from the pseudogene during meiosis. These conversions can involve one (microconversions) or more pseudogene variants (40–42). Differently, 5-10% of CAH alleles observed in most populations are characterized by *CYP21A2* pathogenic variants that do not result in gene conversions (43–45).

**Figure 2:**
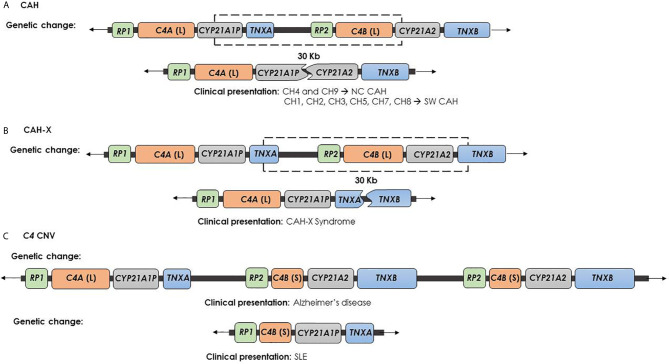
Genetic changes and clinical presentation. **(A)** CAH chimera (*CYP21A1P*/*CYP21A2*) is caused by recombination between *CYP21A1P* and *CYP21A2* and causes the impairment of *CYP21A2* and the deletion of *C4B*, but leaves safe the *TNXB* gene. CH-1, CH-2, CH-3, CH-5, CH-6, CH-7 and CH-8 chimeras are involved in the severe SW form of CAH. In contrast, CH-4 and CH-9 chimeras are generally related to a milder phenotype. **(B)** CAH-X chimera (*TNXA*/*TNXB*) is caused by recombination between *TNXA* and *TNXB* and produces the complete deletion of *CYP21A2* gene, the impairment of *TNXB* gene and the deletion of *C4B*. This contiguous deletion is termed CAH-X and causes CAH-X syndrome. **(C)** Psychiatric and autoimmune diseases due to *C4* CNV: the high *C4B* copy number has been described as Alzheimer’s disease risk factor, while the *C4A* deficiency was related to SLE.

The 20–25% of the cases of 21-hydroxylase deficiency is related to large misalignment due to unequal crossing over during meiosis process. This kind of event may cause gene deletion or amplification, and also broader deletions involving *CYP21A2* gene and the other contiguous genes (40–42). *CYP21A1P*/*CYP21A2* chimeric gene is the result of a recombination between *CYP21A1P* and *CYP21A2* genes, as an unequal crossing over occurs during meiosis. Based on the *C4B* form of the gene, i.e. long or short, the rearrangement results into a 26 or 32 Kb deletion, encompassing the 3′ end of *CYP21A1P*, all of the *C4B* gene, and the 5′ end of the *CYP21A2* gene. This event leads to a single non-functional chimeric gene containing the *CYP21A1P* at the 5’ end and the *CYP21A2* at the 3’ end (**Figure 2A**). To date 9 different chimeric *CYP21A1P*/*CYP21A2* genes have been found and characterized (46–55). In particular, two groups of chimeras, classic and attenuated, have been identified: chimeric genes where the junction site is located downstream of the c.293-13C/A>G mutation in the intron 2 (CH-1, CH-2, CH-3, CH-5, CH-6, CH-7, CH-8) are associated with the severe Salt Wasting form of CAH. In contrast, CH-4 and CH-9 chimeras, carrying the weaker *CYP21A1P* promoter and the sole p.(Pro30Leu) variant, are commonly related to a milder phenotype (47).

Unequal crossover is also the cause of copy number changes of *RCCX* segment. The most well-known case is an haplotypic *RCCX* CNV structure containing three distinct segments with two *CYP21A2* gene copies and one *CYP21A1P* pseudogene copy (56–62). Generally, the *CYP21A2* gene located downstream the *TNXA* gene shows a wild-type nucleotide sequence, or carries one or more deleterious variants. Conversely, the presence of the *CYP21A2* p.(Gln319Ter) mutation characterized the gene copy located next to *TNXB* gene (13, 57–64). To date, 8 different haplotypes with two active *CYP21A2* genes on a chromosome 6 have been detected (63). The absence of a clear correlation between genotype and phenotype observed in many individuals is solved by the existence of these rare haplotypes, underlying the need of the *RCCX* CNV assessment in the molecular diagnosis of 21-hydroxylase deficiency (56, 65, 66).

Finally, the complete deletion of *CYP21A2* gene can occur as the result of an unequal crossing over between *TNXA* and *TNXB* genes. This event produces a chromosome with two copies of *CYP21A2* gene and a chromosome where the arrangement of the *RCCX* segment shows the *C4*-*CYP21A1P*-*TNXA*/*TNXB* sequence, lacking *CYP21A2* gene copy. This condition is associated to the CAH-X Syndrome (67).

### CAH-X Syndrome

Ehlers-Danlos syndromes (EDS) are a clinically and genetically heterogeneous group of heritable connective tissue disorders characterized by joint hypermobility (JH), skin hyperextensibility, and tissue fragility. EDS is typically caused by autosomal dominant mutations in collagen-encoding genes or in genes encoding collagen-modifying enzymes (68). Tenascin-X deficiency causes a clinically distinct form of EDS due to homozygous or compound heterozygous pathogenic variants in the *TNXB* gene. Pathogenic variants account in the coding region of the EGF-like repeats or the fibronectin type III domain of the tenascin protein. The clinical phenotype resembles the classical EDS type with a pattern of autosomal recessive inheritance (69, 70). Heterozygosity for severe *TNXB* mutations causes *TNXB* haploinsufficiency and it is related to hypermobility type EDS (hEDS), characterized by JH, recurring joint dislocations, joint pain and structural cardiac valve abnormality (71). The CAH-X term was first used for the description of a specific subgroup of CAH affected subjects showing an EDS phenotype caused by *CYP21A2* monoallelic deletion extending into the *TNXB* gene (72). The result of this 30 Kb deletion, caused by a recombination event between *TNXA* and *TNXB* genes, is a chimeric *TNXA*/*TNXB* gene (**Figure 2B**) (73). To date, three *TNXA*/*TNXB* chimeras that differ in the junction site and result in a contiguous *CYP21A2* and *TNXB* gene deletion (CH-1 to CH-3) have been reported (72, 74, 75). CAH-X CH-1 is characterized by a *TNXA* pseudogene derived 120-bp deletion in exon 35 that causes the non-functionality of the gene and also results in decreased *TNX* expression in both dermal and serum, claiming an haploinsufficiency mechanism (69, 72). CAH-X CH-2 is characterized by the variant c.12174C>G (p.Cys4058Trp) (exon 40) derived from *TNXA* pseudogene. This substitution deletes a cysteine residue and leads to the loss of a critical disulfide bond in the tertiary structure of the *TNX* C-terminal fibrinogen-like domain (74). The third chimera, termed CAH-X CH-3, has *TNXB* exons 41-44 substituted by *TNXA* and it is characterized by a cluster of 3 closely linked variants also derived from *TNXA* pseudogene: the c.12218G>A (p.Arg4073His) in exon 41 and the c.12514G>A (p.Asp4172Asn) and the c.12524G>A (p.Ser4175Asn) in exon 43 (75). Computational studies showed that the p.(Arg4073His) variant interferes with TNX fibrinogen-like domain stability. In particular, the arginine 4073 is predicted to form a cation-pi interaction with the p.Phe4080 residue, which is lost in the p.(Arg4073His) change, penalizing the folding energy with a loss of 35 kcal/mol. The remaining variants in the cluster did not significantly affect the folding energies in the models (75). Differently to CAH-X CH-1 chimera, CH-2 and CH-3 not reduce the TNX expression but produce altered proteins and are associated with a dominant-negative effect.

All the *TNXA*/*TNXB* chimeras cause EDS in monoallelic or biallelic form regardless of CAH status, although patients with CAH usually show more severe EDS manifestations with respect to carriers without CAH (69, 72, 74–76). Approximately 10% of patients with CAH due to 21-hydroxylase deficiency are affected by CAH-X (74). Recently, Marino et al. reported that the overall prevalence of CAH-X in a large cohort of Argentine CAH patients was 14%, which was similar to that previously found in a large cohort from the National Institutes of Health and in the Chinese population (15% and 14% respectively) (77–79). In addition, Lao et al. reported a particularly high prevalence (29.2%) of CAH-X in 21-hydroxylase deficient patients carrying the 30 kb deletion (78).

Regarding clinical manifestations, CAH-X affected subjects show generalized JH, subluxation and chronic arthralgia, while cardiac abnormalities have been observed in about 25% (80). More severe clinical manifestations were found in patients with a biallelic than in those with a monoallelic form (8, 10). In addition, compared to haploinsufficiency, a dominant-negative effect causes a more severe phenotype displayed by greater skin and joint involvement (74). The diagnosis of EDS due to CAH-X relies mainly on clinical evaluations including physical examination for JH, skin characteristics and imaging. A serum tenascin-X test, based on enzyme-linked immunosorbent assay, has been developed to identify complete deficiency, but it is not accurate in identifying heterozygous forms (69, 81). Molecular diagnosis represents a valid support to the clinical evaluation of CAH-X and, in this context, Sanger sequencing results to be the most reliable an informative method for all *TNXB* variations, even if it is laborious and expensive (82).

### Complement Component *C4* CNV

Complement component *C4* is a central protein in the classical and lectin pathways within the complement system (83). The two isotypes of C4, which differ by only four amino acids, demonstrate differential chemical reactivities: C4A displays higher affinity for amino group-containing antigens or immune complexes, and C4B for hydroxyl group-containing antigens (84, 85). In the general population, the most common *RCCX* haplotype consists of two segments with two C4 in tandem genes coding for *C4A* and *C4B*. So, approximately 60% of healthy individuals have two *C4A* and two *C4B* genes (14, 86, 87). However, deletions and duplications of *C4* genes are well documented and the human *C4* locus has been identified as a functional CNV hotspot within the *RCCX* region. *C4* isotypes involvement is described in several pathological conditions (88). For instance, an high *C4A* gene dosage represents a relevant schizophrenia risk factor, while both *C4A* or *C4B* high copy number is related to Alzheimer’s disease (89, 90) (**Figure 2C**). The presence of one *C4A* or *C4B* gene is called heterozygous *C4A* or *C4B* deficiency, while the presence of no functional *C4A* or *C4B* genes causes complete *C4A* or *C4B* deficiency and is called homozygous *C4* deficiency (14). Homozygous deficiencies of complement *C4A* or *C4B* are detected in 1-10% of populations. Homozygous deficiency of *C4A* has been reported to associate with increased frequency of autoimmune diseases, whereas homozygous *C4B* deficiency has been associated with increased susceptibility of bacterial and enveloped viral infections (91, 92). Many studies support the association between homozygous *C4A* deficiency and systemic lupus erythematosus (SLE) (93–97) (**Figure 2C**).

*C4* structural variations frequently arise in CAH affected subjects with relevant clinical implications, particularly in relation to psychiatric morbidity and autoimmunity (98, 99). Moreover, Lao et al. reported in a cohort of 145 CAH subjects with 21-hydroxylase deficiency, the correlation between *C4A* copy number and the externalization of psychiatric comorbidity (98). Interestingly, authors specified that *C4B* copy number was the determinant of *C4* serum levels in CAH patients because *C4B* copy number varied in CAH patients carrying the 30-Kb deletion and in NC patients carrying the p.(Val282Leu) variant. In fact, as a consequence of 30 Kb deletion, both *C4B* and *CYP21A2* genes are frequently lost concurrently, producing a *CYP21A1P*/*CYP21A2* or *CYP21A1P*-*TNXA*/*TNXB* chimera (**Figures 2A, B**). Conversely, the known association of the NC p.(Val282Leu) variant with high total *C4* copy number was found to be due to a duplication of *C4B* gene, not *C4A* (98, 100).

Recently, Falhammar et al. reported an increased prevalence of autoimmune disorders in a large cohort of Swedish patients with 21-hydroxylase deficiency (99). However, some limitations of the study were point out. In particular, the relatively young age of the patients and the possible protective effects of glucocorticoid treatment may have led to underestimates in the lifetime risks for autoimmune disorders (99).

The complex genetics of human histocompatibility complex provides evidences that *RCCX* genotype being related to *C4* could represent a further risk factor for additional illnesses in CAH affected subjects with 21-hydroxylase deficiency. However, the role of the *C4* gene dosage related to *CYP21A2* genotype in CAH patients needs to further investigations.

## Discussion

*RCCX* CNV represents a complex, multiallelic and tandem CNV in the MHC class III region. Genetic recombination events typically affect this genomic region due to the peculiar co-presence of genes and pseudogenes with high sequence homology, causing frequent misalignment during meiosis. The challenging related to the molecular diagnosis of 21-hydroxylase deficiency, owed to the complexity of the *RCCX* CNV structure, are well documented. For this reason, it is essential to refer to effective guidelines for the standardization of molecular genetic testing of CAH due to *CYP21A2* defects (101). In addition, as recently suggested, including CAH-X chimeras determination in 21-hydroxylase deficiency molecular testing would be particularly beneficial for individuals carrying an allele with the “30Kb deletion”. In fact, a very early CAH-X diagnosis could be offered to young children before hypermobility evaluation is applicable, and to enable early screening for cardiac defects (102). However, a reflection is currently in progress on the need to carry out further studies in order to broader the knowledge and the expertise on CAH-X before including respective methods in routine diagnostic procedures (103, 104).

Finally, novel and larger studies are required in order to elucidate the role of *C4* dosage in several disorders, especially in CAH patients with 21-hydroxylase deficiency.

## Author Contributions

LF and EP researched and wrote a first draft of the review. PC and CC revised the final version of the manuscript. All authors contributed to the article and approved the submitted version.

## Conflict of Interest

The authors declare that the research was conducted in the absence of any commercial or financial relationships that could be construed as a potential conflict of interest.

## Publisher’s Note

All claims expressed in this article are solely those of the authors and do not necessarily represent those of their affiliated organizations, or those of the publisher, the editors and the reviewers. Any product that may be evaluated in this article, or claim that may be made by its manufacturer, is not guaranteed or endorsed by the publisher.
